# Toxicological Evaluation and *In Silico* Identification of Acetylcholinesterase Inhibitors in a Commercial Polyherbal Formulation *(KWAPF01)*

**DOI:** 10.1155/2022/4388941

**Published:** 2022-07-15

**Authors:** A. Raliat Aladodo, Ibrahim O. Ibrahim, Saheed Sabiu

**Affiliations:** ^1^Department of Medical Biochemistry and Pharmacology, Faculty of Pure and Applied Sciences, Kwara State University, Malete, Nigeria; ^2^Department of Biotechnology and Food Science, Durban University of Technology, Durban, South Africa

## Abstract

This study investigated the toxicological implications of a commercial polyherbal formulation, KWAPF01. Twenty-four Wistar rats were randomized into six groups of four animals per group. The animals in Group 1 were administered placebo and designated as control, while the rats in Groups 2 to 6 were administered 1000, 1500, 2000, 2500, and 3000 mg/kg bodyweight single oral dose of KWAPF01, respectively, and subsequently monitored for gross morphological and behavioural changes for 72 h. Piloerection, reduced motility, and tremor were observed in experimental groups, and the median lethal dose (LD_50_) of the extract was 2225.94 mg/kg bodyweight. The 11 compounds identified through HPLC analysis of the extract were docked against acetylcholinesterase (AChE), and the docking scores ranged from −5.3 to −10.8 kcal/mol, with catechol (−5.3 kcal/mol) and berberine (−10.8 kcal/mol) having the highest and lowest binding energies, respectively. Judging by the results, it could be inferred that some of the constituents of KWAPF01 have a direct impact on the nervous system and this is possibly elicited via the cholinergic system as it contains a nicotinic acetylcholine receptors agonist and potential inhibitors of AChE. Therefore, the use of KWAPF01 needs to be cautiously guided.

## 1. Introduction

Traditional medicine is a diverse health practice involving the use of plants, animals, spiritual therapies, and manual techniques used singularly or in combination to maintain good health status as well as to treat, diagnose, or prevent illness [1]. Since antiquity, herbal medicines, an integral part of traditional medicines, have been used in the treatment of diseases [2] and remain an important part of the global healthcare delivery systems, especially in Africa and some Asian countries [3, 4]. The upsurge in the trend of herbal medicine usage as curative medicine is partly related to its cultural acceptability coupled with being relatively more affordable and easily accessible than conventional medicine [5]. Plant materials (roots, leaf, seed, bark, flowers, etc.) have been reported to be used to treat and manage several diseases such as diabetes [6, 7], hypertension [8], and cataracts [9]. Furthermore, several drugs used in conventional medicine were originally derived from plants. For example, salicylic acid, a precursor of aspirin, was first derived from *Salix alba* (white willow) tree bark [10]. Similarly, artemisinin is an antimalarial drug derived from *Artemisia annua* (sweet wormwood), a prominent herb in Chinese traditional medicine [11].

Of recent, however, the increased demand and usage of herbal medicines coupled with reported cases of adverse reactions, especially when used singly, or concurrently with orthodox medicines have raised concerns and fears over the quality, efficacy, and safety of such products [12]. While the components of herbal formulations that may elicit toxic effects could exist as natural components of plants, they may also arise from contaminants acquired during preparation and storage. Also, the indiscriminate, irresponsible, or nonregulated use of herbal medicines may put the health of the users at risk of toxicity. Other factors such as herb-drug interactions, herb-herb interactions, lack of adherence to good manufacturing practices, poor regulatory measures, and adulteration may also lead to adverse reactions [13]. These adverse reactions may manifest as neurotoxicity, hepatotoxicity, nephropathy, cardiomyopathy etc. For example, *Teucrium chamaedrys*, commonly referred to as germander, may cause hepatitis and even liver cirrhosis [14]. Therefore, there is a need for a proper and comprehensive toxicological evaluation of herbal medicine before consumption.

KWAPF01 is a deep brown commercial herbal medicine that is commonly used in Kwara State, Nigeria. It is acclaimed to be effective against hypertension, rheumatoid arthritis, gonorrhoea, and convulsion. Its label indicates that it is made from ginger as well as leaves and roots without explicitly, stating the plants from which they were obtained. It is contraindicated in pregnancy, and the exact duration of use and probable side effects are not known. However, due to the acclaimed therapeutic effects, it has received good patronage over the years, particularly in the southwestern region of Nigeria. However, some of its consumers have reported some adverse reactions with symptoms resembling perturbation of the nervous system such as dizziness, tachycardia, and perspiration. Unfortunately, there is currently no scientific data/information substantiating both the therapeutic and toxicity profile of KWAPF01. It is on this background that the present study was conceptualized and undertaken to evaluate the safety profile of KWAPF01 using computational and *in vivo* experimental models.

Through this research, we intend to bridge the existing research gap on KWAPF01 by investigating if it is toxic and determining the constituents responsible for its toxicity. No scientific report has been published concerning these. The aim of the research was achieved by identifying the bioactive components of the formulation, studying the changes in experimental animals following its administration, establishing its median lethal dose in rats, and studying its effects on the nervous system.

## 2. Materials and Methods

### 2.1. Chemicals

Acetonitrile and ethanol were procured from Sigma-Aldrich (St. Louis, MO, USA). Distilled water was obtained from the Department of Chemistry, Faculty of Pure and Applied Sciences, Kwara State University, Malete, Nigeria. Other chemicals and reagents used were of analytical grade.

### 2.2. Herbal Medicine

Fifteen 50 ml bottles of KWAPF01 used in this study were purchased from a medicine store in Ilorin, Kwara State, Nigeria. Before administration, 500 ml of KWAPF01 was filtered (Whatman No.1 filter paper) and the filtrate was freeze-dried at −54°C and 0.45 mbar.

### 2.3. HPLC Analysis

HPLC analysis was conducted as described by Irondi et al. [8] with some modifications. The HPLC system equipped with a binary solvent delivery module and a diode array detector was used in identifying the secondary metabolites of KWAPF01. The extract (10 g) was dissolved in 20 ml of acetonitrile/methanol (50 : 50) solvent, and after 30 minutes, the solution was transferred into a 25 ml standard flask and made up to 25 ml using the same solvent and then filtered. Exactly 10 *μ*l of the prepared sample was injected into Shimadzu's Nexera MX HPLC system at a controlled flow rate of 1 ml/min. Linear gradient elution was employed using methanol/water (70 : 30) as the mobile phase. Identification of secondary metabolites was performed based on the retention times and the spectra characteristics of peaks with those of reference standards.

### 2.4. *In silico* Evaluation

#### 2.4.1. Acquisition and Preparation of Ligands and Receptors

The X-ray crystal structure of *Mus musculus* AChE (PDB ID: 4B83, resolution: 2.40 Å) [15] was retrieved from the Protein Data Bank (https://www.rcsb.org/). The B3V (N-[2-(diethylamino)ethyl]-3-methoxy-benzenesulfonamide) ligand at the enzyme active sites was saved in the pdb format using untransformed coordinates, while the B chain of the homodimer protein alongside all nonstandard residues in chain A of the receptor was deleted. Loop modelling was conducted to position the missing amino acid residues using modeller v9.25 [16], and the resulting structure was subjected to energy minimization using UCSF Chimera V1.15. Dock Prep tools were used to generate pdbqt files with hydrogen and charges added. On the other hand, the 3D structures of the HPLC-identified compounds and donepezil (a standard AChE inhibitor) were downloaded in simple data format (SDF) from PubChem (https://pubchem.ncbi.nlm.nih.gov). The compounds including the co-crystallized B3V ligand were subsequently optimized in Open Babel algorithm v3.1.1 for energy minimization using MMFF94 force field coupled with the addition of hydrogen atoms. The optimized SDF files were converted to pdbqt format using command lines, specifying Gasteiger charge and pH 7.4 options [17].

#### 2.4.2. Docking Protocol Validation

The prepared B3V ligand, in pdbqt format, was redocked using a grid box with centred at 30.22 Å × 23.87 Å × 12.71 Å and a size of 29.21 Å × 30.00 Å × 25.59 Å for *x*, *y*, and *z* coordinates, respectively. The root mean square deviation (RMSD) was used to assess how close the docking protocol could reproduce the crystallographic binding pose, and this was calculated using AutoDock tools. On superimposing the co-crystallized ligand pose on the predicted pose with the lowest binding energy (−7.4 kcal/mol), the RMSD was found to be 2.042 Å value within the acceptable range of <3.0 Å [18].

#### 2.4.3. Molecular Docking

Molecular docking was done as earlier described by Trott and Olson [19] with some modifications. Alongside a standard drug (donepezil), all KWAPF01 ligands pdbqt were docked against the prepared *Mus musculus* AChE pdbqt structure using AutoDock Vina 1.1.2. The docking parameters were provided in a configuration file. The specified grid box centred at 30.22 Å × 23.87 Å × 12.71 Å, and the box size was 29.21 Å × 30.00 Å × 25.59 Å for *x*, *y*, and *z* coordinates, respectively. Energy range, exhaustiveness, and number of modes were specified to be 3, 8, and 10 kcal/mol, respectively. AutoDock Vina results were visualized using the ViewDock tool in Chimera, and ligand-receptor pdb of poses with the lowest binding energy were generated. Interactions were visualized using Discovery Studio v20.1.0.19295.

#### 2.4.4. Pharmacodynamic and Pharmacokinetic Profiling

The KWAPF01 secondary metabolites were acquired in the simplified molecular input line entry system (SMILES) format. The SMILES notations were compiled, labelled using the MarvinSketch program, and then submitted to the SwissADME server (https://www.swissadme.ch/) to predict some of their possible pharmacodynamic and pharmacokinetic properties [20]. The data generated were exported in comma-separated value (CSV) format, and the BOILED-Egg diagram was also retrieved.

### 2.5. *In Vivo* Experimentation

#### 2.5.1. Experimental Animals and Protocol

The 24 Wistar rats (180 ± 20 g) used in this study were obtained from the Animal Holding Unit of the Department of Biochemistry, Faculty of Life Sciences, University of Ilorin, Ilorin, Nigeria. They were acclimatized to the animal housing conditions (temperature, 25–30°C; 12 hours light/12 hours dark cycle; 40–45% relative humidity) for 7 days and had *ad libitum* access to rat pellets (Top Feeds, Nigeria) and tap water. The research adhered to the Principles of the National Research Council Guide [21] and the National Institute of Health for the Care and Use of Laboratory Animals [22].

#### 2.5.2. Acute Toxicity Evaluation

The acute toxicity evaluation was done following the method described by Eniojukan and Aina [23] with some modifications. To minimize animal use, the sample size was determined using the “resource equation” method [24]. The 24 rats were randomized into six groups of four rats each. Rats in Group 1 designated as control received 1 ml of distilled water, while animals in Groups 2 to 6 were administered 1 ml each of a single oral dose of KWAPF01 at 1000, 1500, 2000, 2500, and 3000 mg/kg bodyweight, respectively. After administration, the animals were monitored for gross morphological and behavioural changes, including locomotor activity, piloerection, ptosis, lacrimation, aggressiveness, convulsion, drowsiness, urination, defecation, and mortality through direct observation as described by Eniojukan and Aina [23]. These observations were made for the first 30 minutes after dosing and periodically during the first 24 h with special attention during the first 4 h and daily thereafter for 3 days. The median lethal dose (LD_50_) was thereafter determined using probit analysis, and the 95% confidence interval was constructed [25]. The mortality data obtained post-72-h treatment was analysed using STATA 16 statistical package, and the generalized linear model analysis was conducted. The number of responses per group (deaths) was used as the response variable, while the log of the dose was used as the explanatory variable. The LD_50_ was then calculated from the fitted model, and the confidence interval was constructed using IBM SPSS, version 21. Based on the LD_50_ value obtained, the test substance was ranked on the Hodge and Sterner toxicity scale [26].

## 3. Results

### 3.1. Phytochemical Analysis and HPLC Profiling

**Figure 1:**
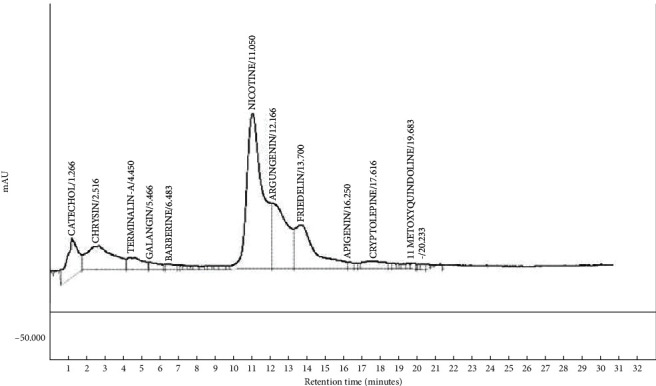
HPLC chromatogram of KWAPF01.

The quantitative phytochemical analysis of the herbal formulation revealed the presence of phenolics, triterpenoids, flavonoids, and alkaloids (Supplementary Table S1). A further probe into its exact constituents through HPLC analysis showed distinct peaks corresponding to 11 compounds (Figure 1 and Table 1). Relative to other identified metabolites, nicotine was the most abundant component of the formulation (41.21%) followed by arjungenin (16.47%) and chrysin (15.22%), while the less abundant metabolites were 11-methoxy-10H-quindoline (0.46%), apigenin (0.59%), and berberine (0.85%) (Figure 1, Table 1).

### 3.2. Acute Toxicity

Table 2 shows the changes observed in the experimental animals following the administration of KWAPF01. There were no observable changes in the control group (Group 1), while piloerection was observed across all groups that received KWAPF01. Tremor and reduced motility were observed at dose 1500 mg/kg bodyweight and above. Data relating to mortality are presented in Supplementary Table S2 and Table 3. The rats dosed at 3000 mg/kg bodyweight died within 72 h, while 75%, 25%, 0%, 0%, and 0% mortality were recorded within the same period at 2500, 2000, 1500, 1000, and 0 mg/kg bodyweight doses.

### 3.3. *In Silico* Studies

#### 3.3.1. Molecular Docking Analysis

Table 4 shows the binding affinity of the ligands for *Mus musculus* AChE. Catechol and nicotine had the lowest binding affinity of −5.3 and −6.7 kcal/mol, respectively, relative to berberine (−10.8 kcal/mol) with a comparable binding affinity score to donepezil (−11.1 kcal/mol), a standard AChE inhibitor, with a binding affinity. Hydrogen bond, *π*-effects, and van der Waal interactions were the predicted interactions.

**Figure 2:**
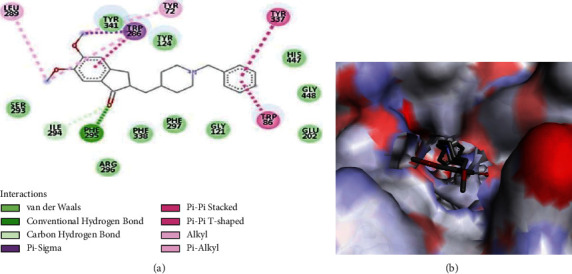
(a) 2D and (b) 3D interaction plots of donepezil with *Mus musculus* AChE.

**Figure 3:**
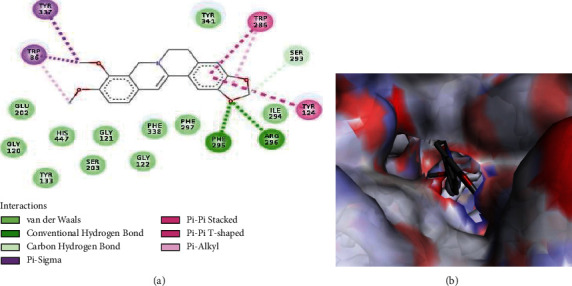
(a) 2D and (b)3D interaction plots of berberine with *Mus musculus* AChE.

The binding poses of donepezil and berberine with the best affinity for AChE are presented in Figures 2 and 3, respectively. Both ligands form van der Waal and pi interactions as well as hydrogen bonds with the amino acid residues at the active site of the enzyme. Trp86, Trp286, Tyr337, and Phe295 are the interacting amino acid residues common to the donepezil-AChE and berberine-AChE interaction plot. The interaction of these residues could be responsible for the observed affinity in each case.

#### 3.3.2. Pharmacodynamic and Pharmacokinetic Profiling

Table 5 shows the selected pharmacodynamic, pharmacokinetic, and physicochemical properties of KWAPF01 secondary metabolites. The compounds have good implicit *n-*octanol/water partition coefficient (iLOGP) values ≤5, molecular weight ≤500 daltons (except arjungenin, which is slightly above 500 daltons), good gastrointestinal (GI) absorption, and 55–56% bioavailability score. Arjungenin, berberine, cryptolepine, terminalin-A, and 11-methoxy-10H-quindoline are P-gp substrates, while berberine, catechol, chrysin, cryptolepine, nicotine, terminalin-A, and 11-methoxy-10H-quindoline are blood-brain barrier permeants, and many of these secondary metabolites are possible inhibitors for some cytochrome (CYP) isoforms (Table 5).

**Figure 4:**
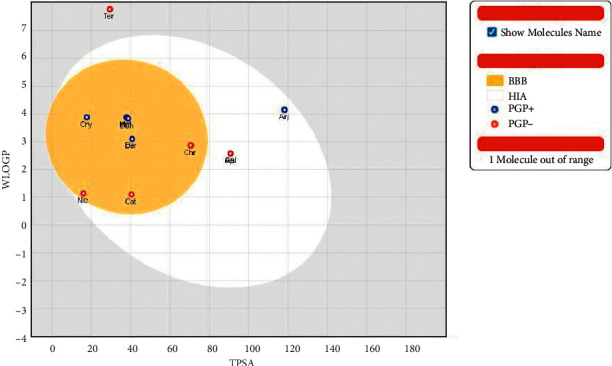
BOILED-Egg diagram of KWAPF01 secondary metabolites. BBB: blood-brain barrier permeant; HIA: passive human gastrointestinal absorption; PGP+: P-glycoprotein substrate; PGP−: non-P-glycoprotein substrate; Cry, cryptolepine; Met, 11-methoxy-10H-quindoline; Ter, terminalin-A; Ber, berberine; Nic, nicotine; Cat, catechol; Chr, chrysin; Api, apigenin; Gal, galangin; Arj, arjungenin; Don, donepezil; Fri, friedelin.

The pharmacokinetic profiles of the secondary metabolites were further represented as BOILED-Egg infographic system (Figure 4), and it was observed that friedelin was out of range, while terminalin-A had poor intestinal absorption. Donepezil and 11-metoxyquindoline share similar WLOGP and TPSA values. Apigenin and galangin also share similar TPSA and WLOGP values. Only nicotine, catechol, and chrysin were predicted not to be a P-gp substrate and BBB permeant and have high passive GI absorption. The detailed pharmacokinetic properties of the identified compounds are presented in Table 5.

## 4. Discussion

The upsurge in the use of herbal medicines in treating diseases calls for more effort towards investigating their potential toxicities as many adverse reactions have been reported following the consumption of herbal products [4].

Judging by the LD_50_ value obtained, KWAPF01 could be said to be slightly toxic as earlier reported by Hodge and Sterner [26]. The choice of studying acetylcholinesterase inhibitory potential of the identified metabolites was informed because of the piloerection, tremor, and lacrimation observed following administration of KWAPF01. These observations are some of the clinical signs of acetylcholinesterase inhibition, as reported by Maksimović et al. [27]. AChE is a cholinergic enzyme found primarily at postsynaptic neuromuscular junctions in muscles and nerves [28]. In contrast to most other neurotransmitters, acetylcholine (ACh) postsynaptic action is not terminated by reuptake and AChE is responsible for hydrolysing ACh to acetic acid and choline to terminate neuronal transmission and signalling in the synapse [29]. Therefore, low AChE activity causes over excitation of nerves, ACh dispersal, and activation of nearby neurons [30].

The result of the HPLC analysis of KWAPF01 is consistent with that of phytochemical analysis where all the 11 identifiable compounds belonged to the four major classes of phytocompounds, including phenolics (catechol), triterpenoids (friedelin, arjungenin, and terminalin-a), flavonoids (chrysin, galangin, and apigenin), and alkaloids (nicotine, berberine, cryptolepine, and 11-methoxy-10H-quindoline). Going by this composition, it could be expected that KWAPF01 will have a broad range of biological activities. For instance, among the health beneficial constituents of the formulation are flavonoids such as apigenin, chrysin, and galangin with anti-inflammatory and antioxidant effects [31, 32]. Arjungenin has been reported to possess hepatoprotective [33] and antiviral activities [34], while cryptolepine has an antihyperglycemic effect [35]. Nevertheless, the results of the acute toxicity studies are consistent with that of the computational analysis, suggesting that KWAPF01 affects the nervous system as it contains nicotine and many molecules with the prospect to inhibit AChE as shown by the molecular docking results. These include berberine, friedelin, galangin, and chrysin. Among the components of KWAPF01, berberine has the highest affinity (lowest binding energy) for AChE. The low binding energy predicted is a result of noncovalent interactions such as hydrogen bond, *π*-effects (e.g., pi-pi and pi-sigma), and van der Waal contacts that it established with the AChE.

The majority of the identified molecules were predicted to have high gastrointestinal absorption except for friedelin and terminalin-A, and this suggests that the contributions of the two molecules to the observed changes could be minute. However, all the compounds had iLOGP (octanol-water partition coefficient) less than 5, and according to Lipinski's rule, they can permeate the lipid bilayer membrane. The bioavailability scores show the probability of the molecule having at least 10% oral bioavailability, and the majority of the identified compounds and donepezil have the same score. However, the P-glycoprotein efflux system found in the GI tract, blood-brain barrier, and several regions of the body [20, 36] could limit the bioavailability of the molecules that were predicted to be P-gp substrates. Except for arjungenin, friedelin, nicotine, and terminalin-A, the compounds found in KWAPF01 could bring about some form of drug-drug interactions when they are administered with other drugs as they all inhibit some significant cytochrome isoforms, which are responsible for most cytochrome biotransformation [37, 38].

Nicotine is the most abundant secondary metabolite in KWAPF01 constituting 41.21% of the herbal formulation. It has been reported to be a potent agonist of nicotinic acetylcholine receptors (nAChRs) except at nAChR*α*9 and nAChR*α*10 subunits [39]. It acts on pentameric nAChRs throughout the nervous system and skeletal muscle and some other non-neuronal sites. The subunits of nAChRs, in tissues, vary and have different characteristics on the binding of agonists [40]. Nicotine binding activates the release of catecholamine leading to ventricular tachycardia [41]. Also, catechol has been reported to cause convulsion, peripheral vasoconstriction, and subsequent increased blood pressure as reported by the United States Environmental Protection Agency [42].

The synergistic effects of KWAPF01 constituents particularly nicotine, catechol, and other molecules (berberine, friedelin, apigenin, chrysin, and galangin) with high affinity for AChE as suggested by the molecular docking results might have aggregated to the behavioural changes, morphological changes, and mortality observed at 2000, 2500, and 3000 mg/kg bodyweight doses during the acute toxicity studies.

## 5. Conclusion

Overall, consequent upon the data presented in this study, it could be inferred that the KWAPF01 commercial formulation has components that can affect the nervous system particularly the cholinergic system, as evident from the acute toxicity and molecular docking results, and this effect is elicited possibly through the inhibition of AChE, a key enzyme of the cholinergic system, and agonistic effect at nAChRs. Based on these observations, it is recommended that consumption of KWAPF01 should be cautiously guided as it contains components with prospects to perturb the nervous systems and those that can make its users addicted. Studies investigating repeated dosing for neurotoxicity of KWAPF01 and the exact mechanism of actions of the identified AChE inhibitors are imperative. It is equally important to establish if the prospective AChE inhibitors identified in this study can be optimized for the treatment of diseases resulting from low levels of acetylcholine, such as Alzheimer's disease. Efforts are underway in these directions [43].

## Figures and Tables

**Table 1:** Secondary metabolites in KWAPF01 formulation.

Peaks	Compounds	Molecular formula	Molecular weight (g/mol)	Retention time	%Peak area
1	Catechol	C_6_H_4_(OH)_2_	110.11	1.27	7.15
2	Chrysin	C_15_H_10_O_4_	254.24	2.52	11.05
3	Terminalin-A	C_30_H_52_O_2_	444.70	4.45	3.10
4	Galangin	C_15_H_10_O_5_	270.24	5.47	1.31
5	Berberine	C_20_H_18_NO_4_^+^	336.36	6.48	0.85
6	Nicotine	C_10_H_14_N_2_	162.24	11.05	41.21
7	Arjungenin	C_30_H_48_O_6_	504.70	12.17	16.47
8	Friedelin	C_30_H_50_O	426.70	13.70	15.22
9	Apigenin	C_15_H_10_O_5_	270.05	16.25	0.59
10	Cryptolepine	C_16_H_12_N_2_	232.29	17.62	2.60
11	11-Methoxy-10H-quindoline	C_16_H_12_N_2_O	248.28	19.68	0.46

**Table 2:** Behavioural and morphological changes following oral dosing with KWAPF01.

Dose (mg/kg)	Observations
0	Normal behavioural repertoire
1000	No changes in motility, and mild piloerection
1500	Reduced motility, tremor, and mild piloerection
2000	Reduced motility, tremor, and piloerection
2500	Marked reduction in motility, tremor, and piloerection
3000	Marked reduction in motility, tremor, and piloerection

**Table 3:** Mortality rates after single oral administration of KWAPF01.

Dose (mg/kg)	Number of rats	Mortality	%Mortality
0	4	0	0
1000	4	0	0
1500	4	0	0
2000	4	1	25
2500	4	3	75
3000	4	4	100

LD_50_ value = 2225.94 mg/kg bodyweight (slightly toxic); 95% confidence interval = 1552.39–2774.81 mg/kg.

**Table 4:** Ligands' binding affinity for *Mus musculus* AChE and amino acids involved in the interaction.

S/N	Compound name	Binding affinity (kcal/mol)	Amino acids interacting with ligands	Number of H-bonds
1	Donepezil	−11.1	Leu289, Tyr341, Trp286, Tyr72, Tyr124, Tyr337, His447, Gly448, Glu202, Trp86, Gly121, Phe297, Phe338, Arg296, Phe295, Ile294, Ser293	2
2	Berberine	−10.8	Trp86, Tyr337, Tyr341, Trp286, Ser293, Tyr124, Ile294, Arg296, Phe295, Phe297, Phe338, Gly122, Gly121, Ser203, His447, Tyr133, Gly120, Glu202	3
3	Friedelin	−10.0	Trp286, Tyr72, Tyr341, His287, Tyr124, Phe297, Ile294, Ser293, Leu289, Glu292, Gln291	1
4	Apigenin	−9.8	Asn87, Ser125, Tyr124, Pro88, Gly121, Trp86, Tyr133, Gly120, Tyr119, Glu202, Ile451, Gly448, Tyr449, His447, Tyr337, Asp74, Tyr72, Val73,	3
5	Chrysin	−9.8	Trp286, Tyr124, Tyr341, Tyr337, His447, Tyr449, Trp86, Gly448, Glu202, Ser203, Gly120, Ala204, Gly121, Gly122, Phe338, Phe297	5
6	Galangin	−9.8	Trp286, Tyr72, Tyr341, His287, Tyr124, Phe297, Ile294, Ser293, Leu289, Glu292, Gln291	3
7	Cryptolepine	−9.5	Trp86, Gly121, Tyr124, Ser125, Tyr72, Asp74, Tyr337, His447, Gly448, Ile451, Glu202, Tyr133, Gly120, Gly126	2
8	11-Methoxy-10H-quindoline	−9.4	Tyr124, Ser125, Tyr337, Trp86, Gly126, Gly121, Leu130, Tyr133, Gly120, Ile451, Glu202, Gly448, His447, Asp74, Tyr72	2
9	Terminalin-A	−8.8	Glu292, His287, Leu289, Gln291, Trp286, Ser293, Gly342, Ile294, Tyr341, Tyr72, Leu76	1
10	Arjungenin	−8.4	Trp286, Tyr72, Tyr341, Glu292, Gln291, Leu289, His287, Ser293, Ile294, Phe295, Phe338, Tyr124, Phe297, Gly342	2
11	Nicotine	−6.7	Tyr124, Phe297, Gly121, Phe338, Gly122, His447, Trp86, Tyr337	0
12	Catechol	−5.3	Trp86, Tyr337, Glu202, Gly121, Gly120, Tyr133, Ile451, Ser203, Gly448, His447	2

**Table 5:** Selected physicochemical and pharmacokinetic profiles of KWAPF01 secondary metabolites.

Molecules	iLOGP	GI absorption	BBB permeant	P-gp substrate	Cytochrome isoform inhibition	Bioavailability score
Donepezil	3.92	High	Yes	Yes	CYP2D6, CYP3A4	0.55
Apigenin	1.89	High	No	No	CYP1A2, CYP2D6, CYP3A4	0.55
Arjungenin	2.71	High	No	Yes	Nil	0.56
Berberine	0	High	Yes	Yes	CYP1A2, CYP2D6, CYP3A4	0.55
Catechol	1.13	High	Yes	No	CYP3A4	0.55
Chrysin	2.27	High	Yes	No	CYP1A2, CYP2D6, CYP3A4	0.55
Cryptolepine	2.46	High	Yes	Yes	CYP1A2, CYP2C19, CYP2D6, CYP3A4	0.55
Friedelin	4.55	Low	No	No	Nil	0.55
Galangin	2.08	High	No	No	CYP1A2, CYP2D6, CYP3A4	0.55
Nicotine	2.14	High	Yes	No	Nil	0.55
Terminalin-A	4.86	Low	No	No	Nil	0.55
11-Methoxy-10H-quindoline	2.46	High	Yes	Yes	CYP1A2, CYP2C19, CYP2D6, CYP3A4	0.55

GI: gastrointestinal; BBB: blood-brain barrier; P-gp: P-glycoprotein; iLOGP: octanol-water partition coefficient.

## Data Availability

The data obtained in this study are available from the corresponding author upon request.

## Supplementary Materials

Supplementary Materials Supplementary Table S1: Quantitative phytochemical composition of KWAPF01 extract. GAE: gallic acid equivalent; QAE: quercetin equivalent; DE: diosgenin equivalent; QE: quinine equivalent; TAE: tannic acid equivalent. Supplementary Table S2: STATA probit LD_50_ analysis output. The LD_50_ was calculated from the model fitted from the output above. *α* = *βx* + *c*, where *α* is the invNorm of response rate (0 at 50%), *β* is the coefficient of response variable (16.5331), *x* is the response variable (log of dose), which is usually the unknown variable, and *c* is the constant (−55.3449). The confidence interval was generated using IBM SPSS, version 21. .

## References

[1] AlRawi S. N., Khidir A., Elnashar M. S. (2017). Traditional Arabic & Islamic medicine: validation and empirical assessment of a conceptual model in Qatar. *BMC Complementary and Alternative Medicine*.

[2] Leonti M., Verpoorte R. (2017). Traditional Mediterranean and European herbal medicines. *Journal of Ethnopharmacology*.

[3] Al-Ghamdi S., Aldossari K., Al-Zahrani J. (2017). Prevalence, knowledge and attitudes toward herbal medication use by Saudi women in the central region during pregnancy, during labor and after delivery. *BMC Complementary and Alternative Medicine*.

[4] Amzat J., Razum O. (2018). *Towards a Sociology of Health Discourse in Africa*.

[5] Mensah M. L., Komlaga G., Forkuo A. D., Firempong C., Anning A. K., Dickson R. A. (2019). Toxicity and safety implications of herbal medicines used in Africa. *Herbal Medicine*.

[6] Aladodo R. A., Balogun E. A., Sunmonu T. O., Nurain I. O. (2017). Combinatorial effects of aqueous root extract of jatropha curcas and J. Gossypiifolia in alloxan-induced diabetic rats. *Iranian Jornal of Toxicology*.

[7] Sulyman A. O., Akolade J. O., Sabiu S. A., Aladodo R. A., Muritala H. F. (2016). Antidiabetic potentials of ethanolic extract of Aristolochia ringens (Vahl.) roots. *Journal of Ethnopharmacology*.

[8] Irondi E. A., Agboola S. O., Oboh G., Boligon A. A., Athayde M. L., Shode F. O. (2016). Guava leaves polyphenolics-rich extract inhibits vital enzymes implicated in gout and hypertension in vitro. *Journal of Intercultural Ethnopharmacology*.

[9] Ajani E. O., Sabiu S., Odufuwa K. T., Ibrahim T. B., Salau B. A. (2016). Evaluation of lens aldose reductase inhibitory and free radical scavenging potential of fractions of Lonchocarpus cyanescens: potential for cataract remediation. *Pharmacognosy Journal*.

[10] Shara M., Stohs S. J. (2015). Efficacy and safety of white willow bark (Salix alba) extracts. *Phytotherapy Research*.

[11] Shi P., Fu X., Liu M. (2017). Promotion of artemisinin content in Artemisia annua by overexpression of multiple artemisinin biosynthetic pathway genes. *Plant Cell, Tissue and Organ Culture*.

[12] Komolafe K., Komolafe T. R., Fatoki T. H. (2021). Coronavirus disease 2019 and herbal therapy: pertinent issues relating to toxicity and standardization of phytopharmaceuticals. *Revista Brasileira De Farmacognosia*.

[13] Sammons H. M., Gubarev M. I., Krepkova L. V. (2016). Herbal medicines: challenges in the modern world. Part 2. European Union and Russia. *Expert Review of Clinical Pharmacology*.

[14] Brown A. C. (2017). Liver toxicity related to herbs and dietary supplements: online table of case reports. Part 2 of 5 series. *Food and Chemical Toxicology*.

[15] Andersson C. D., Forsgren N., Akfur C. (2013). Divergent structure-activity relationships of structurally similar acetylcholinesterase inhibitors. *Journal of Medicinal Chemistry*.

[16] Šali A., Blundell T. L. (1993). Comparative protein modelling by satisfaction of spatial restraints. *Journal of Molecular Biology*.

[17] O’Boyle N. M., Banck M., James C. A., Morley C., Vandermeersch T., Hutchison G. R. (2011). Open Babel: an open chemical toolbox. *Journal of Cheminformatics*.

[18] Debnath P., Debnath B., Bhaumik D., Debnath S. (2020). In silico identification of potential inhibitors of ADP‐ribose phosphatase of SARS‐CoV‐2 nsP3 by combining E‐pharmacophore‐ and receptor‐based virtual screening of database. *Chemistry Select*.

[19] Trott O., Olson A. J. (2009). AutoDock Vina: improving the speed and accuracy of docking with a new scoring function, efficient optimization, and multithreading. *Journal of Computational Chemistry*.

[20] Ahmad J. B., Ajani E. O., Sabiu S. (2021). Chemical group profiling, in vitro and in silico evaluation of aristolochia ringens on *α*-amylase and *α*-glucosidase activity. *Evidence-based Complementary and Alternative Medicine*.

[21] National Research Council (Nrc) (2011). *Guide for the Care and Use of Laboratory Animals*.

[22] National Institute of Health (NIH) (1985). *Care and Use of Laboratory Animals*.

[23] Eniojukan J., Aina B. (2010). Toxicological profiles of commercial herbal preparation, Jobelyn. *International Journal of Health Research*.

[24] Jatinder S. (2012). National centre for replacement, refinement, and reduction animals in research. Experimental design/statistics. *Journal of Pharmacology and Pharmacotherapy*.

[25] Finney D. J. (1952). *Probit Analysis: A Statistical Treatment of the Sigmoid Response Curve*.

[26] Hodge A., Sterner B. (2005). *Toxicity Classes*.

[27] Maksimović Ž. M., Duka D., Bednarčuk N., Škrbić R., Stojiljković M. P. (2021). Onset rate and intensity of signs of organophosphate poisoning related to paraoxon dose and survival in rats. *Scripta Medica*.

[28] Rotundo R. L. (2020). Acetylcholinesterase at the neuromuscular junction. *Neuroscience Letters*.

[29] Adeyinka A., Kondamudi N. P. (2018). *Cholinergic Crisis*.

[30] Reale M., Costantini E. (2021). Cholinergic modulation of the immune system in neuroinflammatory diseases. *Diseases*.

[31] Zeinali M., Rezaee S. A., Hosseinzadeh H. (2017). An overview on immunoregulatory and anti-inflammatory properties of chrysin and flavonoids substances. *Biomedicine & Pharmacotherapy*.

[32] Lee H. N., Shin S. A., Choo G. S. (2018). Anti‑inflammatory effect of quercetin and galangin in LPS‑stimulated RAW264.7 macrophages and DNCB‑induced atopic dermatitis animal models. *International Journal of Molecular Medicine*.

[33] Wang G., Wang G. K., Liu J. S., Yu B., Wang F., Liu J. K. (2010). Studies on the chemical constituents of Kalimeris indica. *Zhong yao cai = Zhongyaocai = Journal of Chinese Medicinal Materials*.

[34] T T., T A., P F., FathimaKhanum F. (2020). In-silico therapeutic investigations of arjunic acid and arjungenin as an FXR agonist and validation in 3T3-L1 adipocytes. *Computational Biology and Chemistry*.

[35] Ameyaw E. O., Asmah K. B., Biney R. P. (2018). Isobolographic analysis of co-administration of two plant-derived antiplasmodial drug candidates, cryptolepine and xylopic acid, in *Plasmodium berghei*. *Malaria Journal*.

[36] Turner A. P., Alam C., Bendayan R. (2020). Efflux transporters in cancer resistance: molecular and functional characterization of P-glycoprotein. *Drug efflux pumps in cancer resistance pathways: from molecular recognition and characterization to possible inhibition strategies in chemotherapy*.

[37] Sychev D. A., Ashraf G. M., Svistunov A. A. (2018). The cytochrome P450 isoenzyme and some new opportunities for the prediction of negative drug interaction in vivo. *Drug Design, Development and Therapy*.

[38] Sabiu K., Idowu K. (2022). An insight on the nature of biochemical interactions between glycyrrhizin, myricetin and CYP3A4 isoform. *Journal of Food Biochemistry*.

[39] Silva A. R., Grosso C., Delerue-Matos C., Rocha J. M. (2019). Comprehensive review on the interaction between natural compounds and brain receptors: benefits and toxicity. *European Journal of Medicinal Chemistry*.

[40] Benowitz N. L., Burbank A. D. (2016). Cardiovascular toxicity of nicotine: implications for electronic cigarette use. *Trends in Cardiovascular Medicine*.

[41] Schumer A., Contag S. (2020). Catecholaminergic polymorphic ventricular tachycardia in pregnancy: a case report. *Journal of Medical Case Reports*.

[42] United States Environmental Protection Agency (2016). *Catechol (Pyrocatechol)*.

