# Supplementary material for: Toxicological Evaluation and In Silico Identification of Acetylcholinesterase Inhibitors in a Commercial Polyherbal Formulation (KWAPF01)
Source: Evid Based Complement Alternat Med. 2022 Jul 15;2022:4388941. doi: 10.1155/2022/4388941 (PMC9307333; doi:10.1155/2022/4388941)
Supplement: Supplementary Materials — Supplementary Table S1: Quantitative phytochemical composition of KWAPF01 extract. GAE: gallic acid equivalent; QAE: quercetin equivalent; DE: diosgenin equivalent; QE: quinine equivalent; TAE: tannic acid equivalent. Supplementary Table S2: STATA probit LD50 analysis output. The LD50 was calculated from the model fitted from the output above. α = βx + c, where α is the invNorm of response rate (0 at 50%), β is the coefficient of response variable (16.5331), x is the response variable (log of dose), which is usually the unknown variable, and c is the constant (−55.3449). The confidence interval was generated using IBM SPSS, version 21. . [file 4388941.f1.docx]

Supplementary Table S1: Quantitative phytochemical composition of KWAPF01 extract

| **Phenolic (GAEmg/100g)** | | **Flavonoids (QAEmg/100g)** | **Saponin (DE mg/100g)** | **Alkaloids (QE mg/100g)** | **Tannin (TAE mg/100g)** | |
| --- | --- | --- | --- | --- | --- | --- |
| 100.30 | 84.67 | | 34.05 | 13.49 | | 1.77 |

GAE: Gallic acid equivalent; QAE: quercetin equivalent; DE: Diosgenin equivalent; QE: quinine equivalent; TAE: tannic acid equivalent;

Supplementary Table S2: STATA probit LD50 analysis output


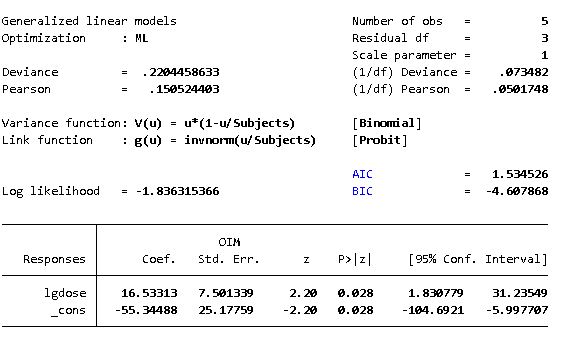


The LD_50_ was calculated from the model fitted from the output above

α=βx + c where α is; the invnorm of response rate (0 at 50%), β is the coefficient of response variable (16.5331), x is the response variable (log of dose) which is usually the unknown variable, and c is the constant (-55.3449). The confidence interval was generated using IBM SPSS version 21.
